# Transperineal ultrasound shear-wave elastography is a reliable tool for assessment of the elastic properties of the levator ani muscle in women

**DOI:** 10.1038/s41598-021-95012-8

**Published:** 2021-07-30

**Authors:** Bertrand Gachon, Xavier Fritel, Fabrice Pierre, Antoine Nordez

**Affiliations:** 1 Department of Obstetrics and Gynecology, Poitiers University Hospital, 2, rue de la Miletrie, 86000 Poitiers, France; 2 Université de Nantes, Mouvement – Interactions – Performance, MIP, EA4334, 44000 Nantes, France; 3 INSERM CIC 1402, Poitiers University, Poitiers University Hospital, Poitiers, France; 4 Institut Universitaire de France (IUF), Paris, France

**Keywords:** Muscle, Urinary incontinence

## Abstract

Our main objective was to assess the intraoperator intersession reproducibility of transperineal ultrasound Shear Wave Elastography (SWE) to measure the levator ani muscle (LAM) elastic properties. Secondary objective was to compare reproducibility when considering the mean of three consecutives measurements versus one. In this prospective study involving non-pregnant nulliparous women, two visits were planned, with a measurement of the shear modulus (SM) on the right LAM at rest, during Valsalva maneuver and maximal contraction. Assessments were done with a transperineal approach, using an AIXPLORER device with a linear SL 18–5 (5-18 MHz) probe. For each condition, 3 consecutive measures were performed at each visit. The mean of the three measures, then the first one, were considered for the reproducibility by calculating intraclass correlation coefficient (ICC), and coefficient of variation (CV). Twenty women were included. Reproducibility was excellent when considering the mean of the 3 measures at rest (ICC = 0.90; CV = 15.7%) and Valsalva maneuver (ICC = 0.94; CV = 10.6%), or the first of the three measures at rest (ICC = 0.87; CV = 18.6%) and Valsalva maneuver (ICC = 0.84; CV = 19.9%). Reproducibility was fair for measurement during contraction. Transperineal ultrasound SWE is a reliable tool to investigate LAM elastic properties at rest and during Valsalva maneuver.

## Introduction

Pelvic floor disorders (PFDs) are frequently occurring conditions, and up to 20% of women experience PFDs during their lifetime^1^. Although the pathophysiology of PFDs is complex, some of these disorders may be explained by vaginal delivery, which can induce pelvic floor damage involving the levator ani muscle (LAM) and/or anal sphincter in up to 15% of women^2,3^. This is supported by data reporting that LAM avulsion is associated with an increased levator hiatus area, which is a primary risk factor of pelvic organ prolapse^3,4^. However, the individual pathophysiology of pelvic floor damage itself remains poorly understood, and safe strategies for identifying high-risk women based on their intrinsic characteristics are desperately required^2^.

Studies using animal models and/or ex vivo human tissue analysis have suggested that since the elastic properties of pelvic floor muscles are the first to change during pregnancy, these elastic properties may be associated with PFD^2,5,6^. The current main limitation is that information about the human mechanical behavior of pelvic floor muscles in vivo is lacking. Indeed, until recently, ex vivo and destructive biomechanical analysis was required to characterize muscle’s mechanical behavior. Therefore, while the literature suggests an association between pelvic floor muscles (and especially the main one, the LAM) and obstetrical pelvic floor damage and /or pelvic floor disorders, this hypothesis remains to be tested in vivo^7,8^. Thus, one of our current challenges is to identify innovative techniques for the assessment of pelvic floor elastic properties to better understand the potential role of these properties in the occurrence of obstetric pelvic floor damage and/or PFD. Furthermore, this technique must be reliable to be implemented in both research designs and clinical practices. The viscoelastic properties of pelvic floor muscles could be measured using a speculum combined with sensors, i.e., a vaginal probe able to measure the force exerted by the pelvic floor muscles on it^9,10^. However, they require an intravaginal intrusive examination, and it remains an indirect measurement on pelvic floor muscles elastic properties (based on the force exerted on the probe/speculum)^2,11^.

Our study focused on a direct quantitative assessment of muscle elasticity using ultrasound shear-wave elastography (SWE). In this technique, a mechanical perturbation was generated using ultrasound to induce the propagation of a shear wave along the main axis of the ultrasound probe^12–14^. The speed of the wave’s propagation was correlated to the shear modulus and the stiffness of the tissue, since the stiffer the tissue, faster was the wave’s propagation^12–14^. This technique seems relevant compared to static or quasistatic elastography because allows a direct quantitative measurement of muscles elastic properties (without the interposition of a standoff pad). In addition, the measurement can be made along the muscle fiber direction, while static elastography measures the transverse behavior (i.e., hardness) that is probably less physiologically relevant^14–17^. Furthermore, SWE is feasible with a superficial linear probe offering the possibility of a transperineal approach for investigating the LAM using the technique of Dietz et al. without any intravaginal examination^18^.

In a previous report we described the feasibility of assessing the elastic properties of LAM in women, using SWE with a transperineal approach. In the present study, we investigate inter-day and intra-operator reliability for LAM to validate its use in future prospective studies. We also investigate the intrasession reliability to check if the procedure could be simplified by doing only one single measure instead of 3 consecutives measures^11^. So, the main objective of the present study was to assess the intraoperator intersession reproducibility of ultrasound SWE to measure the LAM elastic properties in women^12^. The secondary objectives were as follows: (i) to investigate the intrasession reproducibility and (ii) to compare intersession reproducibility when considering the mean of three consecutive measurement versus one single measurement.

## Material and methods

### Study settings

This prospective monocentric study was conducted in our University Department of Obstetrics and Gynecology from July 2019 to August 2020. In the protocol, the time interval between two visits ranged from 12 h to 7 days.

### Population

Eligible participants were non-pregnant, nulliparous women attending a visit to our Gynecology unit. The exclusion criteria were as follows: history of previous delivery (vaginal or cesarean section), personal history of PFD, obese women with a body mass index (BMI) higher than 35 kg·m^−2^, women with muscular disease, women requiring admission to a psychiatric unit, women under judicial protection, and those who were unable to understand French language.

### Data collection

#### Participant characteristics

At the first visit, the participants’ age, height, and weight were collected, and their BMI was calculated.

#### Shear wave elastography assessments

The evaluation protocol during the two visits was similar: ultrasound SWE assessment of the LAM at rest, during subjective maximal Valsalva maneuver, and during subjective maximal perineal contraction. For subjective maximal Valsalva maneuver, it was required from the women to take a deep breath and to push down as much as possible with a closed glottis. This will highly increase the intraabdominal pression and so will induce a cranio-caudal descent of pelvic organs leading to a distension of the levator hiatus with a lengthening of the LAMs. It can be considered as a lengthening of the LAM that should induce an increase in shear modulus^14^. This is in accordance with the childbirth condition because the effort required from the mother is the same and that the same phenomena of LAM lengthening that occurs at childbirth even if the strain magnitude is much higher. This is also in accordance with pelvic floor disorders because the occurrence of pelvic organ prolapse is associated to an overlengthening of the LAMs when intraabdominal pressure increases leading to a prolapse of pelvic organs through it. For the subjective maximal contraction, it was required from the women to contract and tighten her perineum as much as she can. It is also in accordance with the effort performed during physiotherapy procedures. This is an important part of pelvic floor disorders management. Finally, the rest position represents the condition with the lowest load to estimate the intrinsic resting elastic properties of the LAM. For each condition (rest, Valsalva maneuver, and maximal contraction), three consecutive measurements were performed. All measurements during both visits were performed by a single operator, a senior urogynecologist (BG, the first author) with a special interest in pelvic floor imaging. We chose to consistently obtain ultrasound measurements on the right side of the participants based on the convenience of the operator, who was at the right when the participant was in the supine position, and to standardize the procedure.

The principles of ultrasound SWE and the procedure for measuring muscle elastic properties have been widely described and illustrated in previous publications, and specific aspects pertaining to the LAM are described below^11–14^.

**Figure 1:**
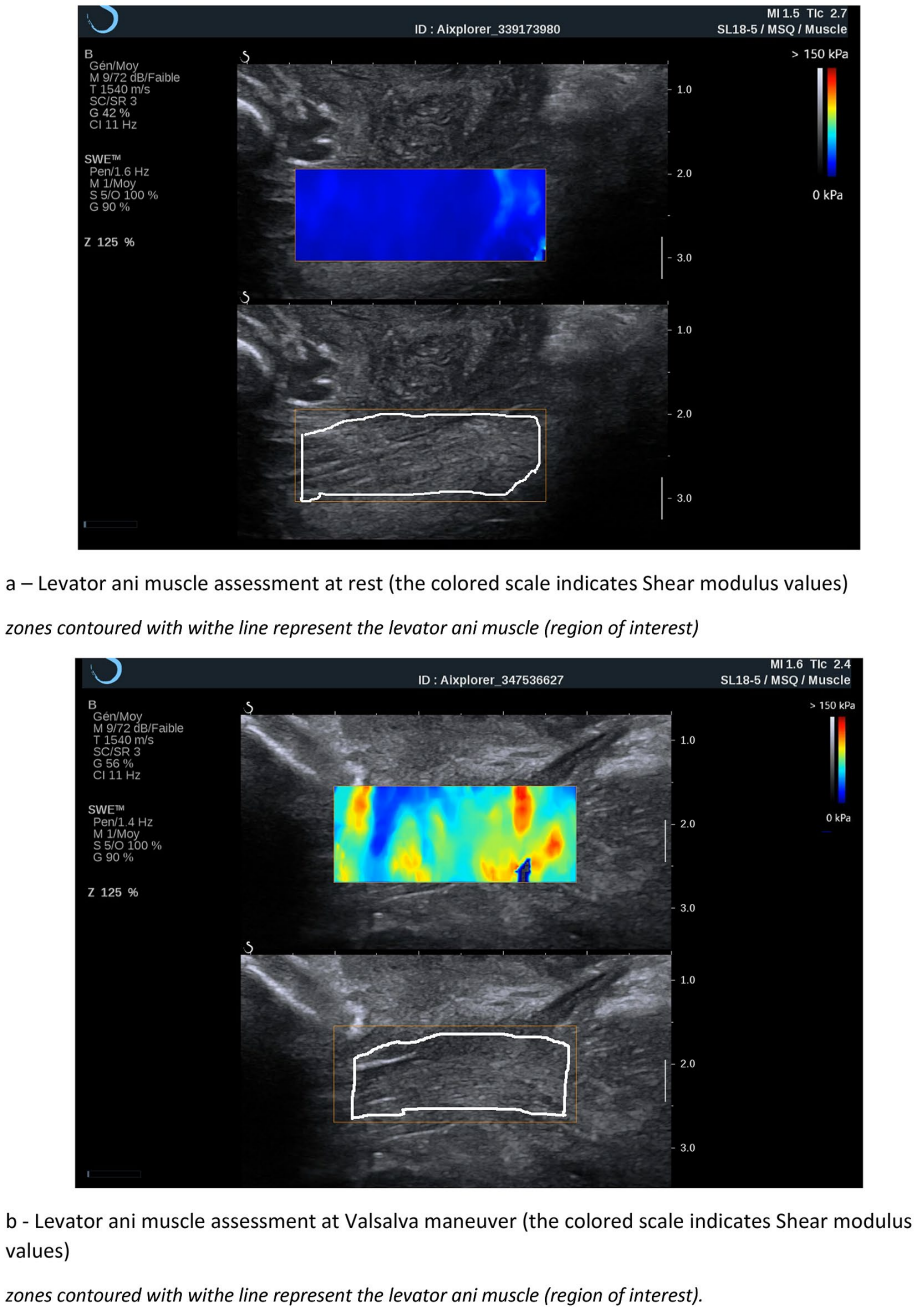
Assessment of the right *levator ani* muscle at rest (**a**) and Valsalva maneuver (**b**).

For measurements in each condition, the participants lay down in the lithotomy position with an empty bladder. The pubic insertion of the right LAM was identified in B-mode ultrasound with a transperineal approach using the procedure reported by Dietz et al., after which we proceeded to perform the SWE acquisition^11,12,18^. Before any LAM assessment, the participants performed 2 initial Valsalva maneuvers with biofeedback, in which visible pelvic floor displacements on the B-mode image were shown to the participant on the screen, to prevent LAM coactivation^19^. For assessments at rest, the participant was asked to relax as much as possible. For assessment during the Valsalva maneuver, the participant was requested to perform a maximal Valsalva maneuver for at least 5 s. For assessment during subjective maximal contraction, the participant was asked to contract her perineum as if she wanted to avoid gas leakage for at least 5 s. Figure 1 shows the LAM assessment at rest (a) and in the subjective maximal Valsalva maneuver (b).

Ultrasound measurements were performed using an AIXPLORER device (V12, SUPERSONIC IMAGINE, France) with a linear SL 18–5 probe (5–18 MHz). As reported above, the muscle location was assessed in B-mode, after which we performed SWE acquisition in a 5-s video clip. Shear modulus (SM) values were averaged over this period. The clip was obtained to limit the influence of inevitable temporal changes (5%)^20^. To provide an idea of the temporal changes during measurements, a video clip of an ultrasound SWE assessment of the LAM during the Valsalva maneuver is provided as online supplementary material (Supplementary material 1).

### Data analysis and statistics

The region of interest was identified and contoured manually using Matlab scripts (The Mathworks, Inc., 2016). For assessments at rest and during the Valsalva maneuver, the mean SM for the whole acquisition was considered. For assessments during subjective maximal perineal contraction, the maximal SM for the acquisition was considered. In case of limited region within the measurement is not possible, the software automatically excludes it for the analysis. As mentioned in a previous publication, the AIXPLORER device provides a measurement of the Young’s modulus that is valid for isotropic tissues. Since muscles are transverse isotropic tissues, the SM was measured by dividing the Young’s modulus by 3^11,12,14,21,22^. In a material, the measure shear wave speed (Vs) along a given direction could be converted to a shear modulus (mu) along this direction thanks to this equation: mu = rho Vs^2^. Considering an incompressible isotropic material, mu (or Vs) could be converted to the Young’s modulus (E): E = 3mu = 3 rho Vs^2.^ In a muscle, since it is not an isotropic material, E is not equal to 3 mu anymore. However, the relationship mu = rho Vs^2^ remains valid. The AIXPLORER directly provides E = 3 rho Vs^2^ (wrong equation for muscles). Therefore, for muscles, it is recommended to divide the values by 3 to assess the shear modulus rather than the Young’s modulus. Dividing the Young’s modulus values by 3 is equivalent to directly consider mu = rho Vs^2^^14,21,22^.

We first described our population in terms of age, mean BMI, and the interval between the two assessments. Continuous variables were reported as mean and standard deviation, and categorical variables by numbers and percentages. On the basis of our primary objective, we analyzed the intersession reproducibility for each mode of assessment (rest, Valsalva, contraction), with the intraclass correlation coefficient (ICC), the standard error of measurement (SEM), and the coefficient of variation (CV) serving as the main judgment criteria. For this analysis, we considered the mean of the three consecutive measurements performed in each session for the analysis. We computed the ICC with 95% confidence intervals for each assessment and calculated the CV^23^. Bland–Altman plots were built according to the methods reported in the original publication^24,25^. Regarding the ICC value we therefore considered that the reliability was excellent if 0.90 or higher, good if between 0.75 and 0.89, moderate if between 0.50 and 0.74 and poor if lower than 0.50^23^.

To address our secondary objectives, we investigated the intrasession reproducibility within three consecutive measurements by using the same methods as for the primary objective: ICC, SEM, and CV. ICC values were interpreted as reported above. We then compared the reproducibility performance when considering the mean of the three measurements or the first of the three consecutive measurements. A priori power calculation was not performed. Considering other studies reporting reliability analysis for ultrasound SWE in peripheral muscles, a study population of 20 women appears to be sufficiently effective^20^.

Statistical analysis was performed using the STATA software (version V14IC; Stata Corporation, College Station, TX, USA). For all analyses, significance was considered for p < 0.05.

### Ethical and reglementary considerations

The study was approved by an ethics committee *(Comité de Protection des Personnes Ile de France 8*, ethical committee for human protection from Ile de France*)* on the 16/07/2018 and is referenced with the ID RCB: 2018-A01422-53. The study was registered on https://clinicaltrials.gov (NCT03602196) on the 26/07/2018. All methods were carried out in accordance with relevant guidelines and regulations. Written and informed consent was obtained from all subjects before any investigation.

## Results

Twenty women were included in this study. Their mean age was 23 years (SD = 4 years) with a mean BMI of 22.6 kg·m^−2^ (SD = 3.2 kg·m^−2^). The mean interval between the two visits was 46.6 h (SD = 39.6 h; range, 24–166 h). All included women completed the study protocol.

**Figure 2:**
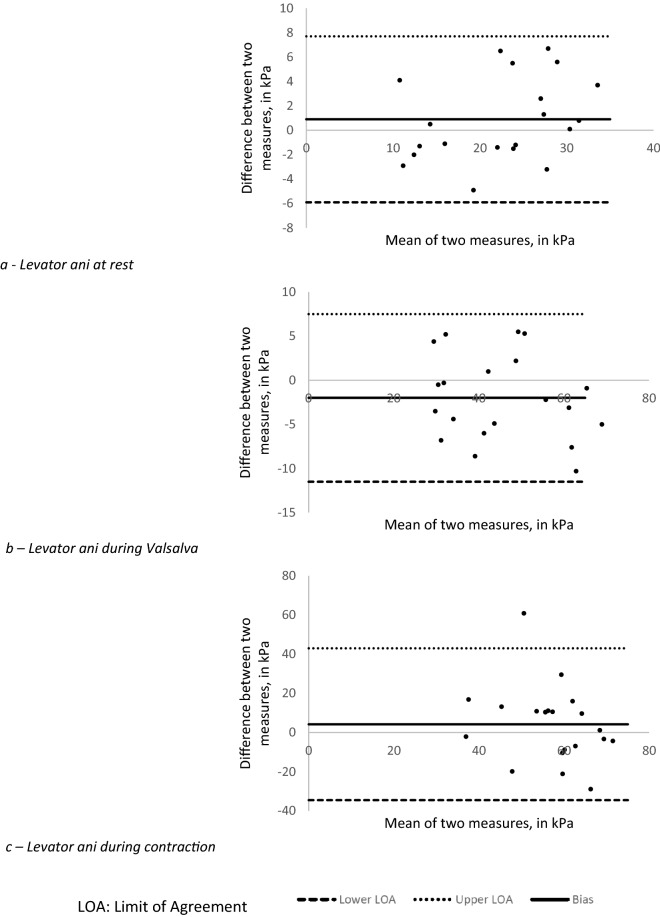
Bland–Altman plots of agreement between V1 (first visit) and V2 (second visit) for the mean levator ani muscle’s shear modulus assessment at each visit and each condition.

In our main analysis, the ICC was excellent for the intersession reproducibility, considering the mean of the three measures at rest and during the Valsalva maneuver (Table 1). Conversely, ICC was poor for measurements performed during subjective maximal contraction (Table 1). Bland–Altman plots are shown in Fig. 2. The results for intrasession reproducibility are reported in Table 2, and they show good reliability at rest and during the Valsalva maneuver, but moderate during subjective maximal contraction.

**Table 1:** Intersession reproducibility performances for the assessment of the right levator ani muscle’s shear modulus.

	Mean shear modulus at V1, in kPa (SD)	Mean shear modulus at V2, in kPa (SD)	ICC [95% CI]	CV, in %	SEM, in kPa
**Intersession reproducibility performances by considering the mean of the 3 measures at each visit**					
Rest	22.8 (8.0)	21.9 (6.8)	0.90 [0.80–0.95]	15.7	3.5
Valsalva	44.5 (13.1)	46.5 (14.2)	0.94 [0.88–0.97]	10.6	4.8
Contraction	59.3 (11.8)	55.1 (15.7)	0.43 [0.07–0.69]	25.1	14.8
**Intersession reproducibility performances by considering one single measure at each visit**					
Rest	22.2 (8.3)	22.0 (7.0)	0.87 [0.74–0.94]	18.6	4.1
Valsalva	43.2 (13.1)	44.2 (16.1)	0.84 [0.68–0.92]	19.9	8.7
Contraction	60.2 (12.0)	56.2 (16.8)	0.61 [0.31–0.80]	22.9	13.3

ICC: Intraclass correlation coefficient, CI: confidence interval, CV: coefficient of variation, SEM: standard error of measurement, V1: first visit, V2: second visit.

**Table 2:** Intrasession reproducibility performances for the assessment of the right levator ani muscle’s shear modulus with 3 consecutive measures.

	1st measure mean shear modulus, in kPa (SD)	2nd measure mean shear modulus, in kPa (SD)	3rd measure mean shear modulus, in kPa (SD)	ICC [95% CI]	CV, in %	SEM, in kPa
Rest	22.1 (7.6)	22.7 (8.4)	22.2 (7.8)	0.84 [0.75–0.89]	21.1	4.7
Valsalva	43.7 (14.5)	46.9 (13.5)	46.8 (15.6)	0.88 [0.75–0.91]	16.6	7.6
Contraction	58.2 (14.6)	61.4 (14.9)	57.2 (15.5)	0.70 [0.57–0.80]	20.2	11.9

ICC: Intraclass correlation coefficient, CI: confidence interval, CV: coefficient of variation, SEM: standard error of measurement.

In Table 1, we report the reproducibility performance indicators for both analyses when considering the mean of the three measurements for each visit and when considering the first of the three consecutive measurements. ICC and other reliability indicators were higher when considering the mean of the three measurements for the rest and Valsalva maneuver measurements (Table 1). Reliability during subjective maximal contraction was poor, regardless of whether we used the mean or the first measurement alone.

## Discussion

### Main results

The intersession reproducibility of ultrasound SWE for measuring the elastic properties of the LAM was excellent at rest and during the Valsalva maneuver but poor during subjective maximal contraction. The reproducibility performance of the mean of three consecutive measurements for each session was higher than that of the first of the three consecutive measurements.

### Justification of methodological choices

We chose the ultrasound SWE method since it is allowing non-invasive and quantitative assessment of the pelvic floor muscles. We have previously reported the feasibility of measuring LAM elastic properties without difficulties, supporting our choice to focus on this approach in the present study^12^. We systematically investigated the right LAM to ensure operator convenience (since the operator was usually on the right side of the supine participant). This approach appears safe since we only included nulliparous women, thereby avoiding women with levator avulsion. Furthermore, a previously feasibility pilot study reported no differences in the SM measured on the right versus left LAM^12^. We considered BMI higher than 35 kg·m^−2^ as an exclusion criterion because measurements for women with very high BMIs could not be performed due to loss of LAM visibility in B-mode ultrasound during the Valsalva maneuver^12^. Finally, for the main analysis, we chose to consider the mean of three consecutives measurements performed at each visit instead of a direct single measurement. We hypothesized that reproducibility will probably be better with this approach since it is difficult to standardize a lithotomy position and even more difficult to standardize a Valsalva maneuver.

### Strengths and Limitations

The main strength of this study is that it provides data about an innovative approach to investigate the elastic properties of pelvic floor muscles with a non-invasive approach that will be much more acceptable for women than other techniques using vaginal speculums or vaginal ultrasound probes^9,26–28^.

The primary limitation of this study is that we only reported intra-operator reproducibility data. This was due to the lack of an additional available experimenter, and because we aimed to use only one experimenter in our projects^11^. However, measurements performed by two experimenters may show specific interoperator discrepancies, and the interoperator reliability will have to be determined by groups that aim to have more than one experimenter in their protocol.

In this study, we made measurement of the mean shear modulus for the largest visible muscle region. Indeed, the viscoelastic properties of a tissues may differ according to the region. This might be true for the muscle which is generally stiffer near to its insertion. Therefore, the good reliability reported in the present study suggest that we were able to reproduce the measurements in a similar region and it is probably a criterion to get reliable measurements. We chose to measure the shear modulus in one single area because, in a clinical view, the part of the LAM accessible with such a transperineal approach is considered as the pubic insertion of the LAM (the one affected by obstetric perineal trauma) and it would not have been clinically justified to perform several measures in different areas.

Another limitation is the standardization of the LAM SWE measurement. Indeed, we only required the participants to lie down in the lithotomy position with an empty bladder without any measurement of thigh opening. This is particularly true for the subjective maximal contraction condition, where the intensity of the contraction was not controlled, since the measurement was highly dependent on the contraction level^29^. Thus, the conditions across measurements may not have been exactly comparable. However, this was a voluntary choice because we aimed to assess the reproducibility in *real-life* conditions since we aimed to perform such measurements in a clinical environment with pregnant women.

Lastly, we did not report any clinical examinations related data and so were not able to investigate the correlation between elastography considerations and clinical observations. Such an analysis is ongoing in a prospective study in pregnant women (ELASTOPELV)^11^.

### Interpretation

We reported excellent reproducibility for assessments performed at rest and with the Valsalva maneuver. Only one previous report has described such a reproducibility analysis of LAM assessment using a transperineal approach, but that study used an abdominal curved probe. In that study, the authors reported good reproducibility of intra-operator intersession assessments at rest (ICC = 0.86 [0.58–0.95]) and during the Valsalva maneuver (ICC = 0.79 [0.54–0.91]), and they did not report measures during contraction^30^.

In our results, the reliabilities at rest and during the Valsalva maneuver were excellent both when considering the mean of the three consecutive measurements and when considering only the first of the three consecutive measurements. This observation would have been the same if we had considered the second or third of the three measurements since the intra-operator intra-session reproducibility was good among these three measurements. This result is interesting and may have direct applications. On the basis of this result, it appears safe to perform a single measurement of the LAM using transperineal ultrasound SWE when the objective is to assess the elastic properties of this muscle at a specific and unique time. If the technique is used to investigate changes across time, it is probably safer to perform three measurements and to consider their mean for the analysis to increase the sensitivity of the examination.

Mean shear modulus for assessment during Valsalva maneuver and contraction were within the same range of values. This could be surprising because, in skeletal muscles, increases in shear modulus are much bigger during contractions^29^ compared to passive lengthening^31^. A first explanation would have been a contraction of the LAM during the Valsalva maneuver that would have led to overestimate the stiffness of the muscle in this condition. This was probably not the case because we systematically took care of avoiding any LAM coactivation during Valsalva maneuver thanks to bio feedback procedures as recommended by Orno et al.^19^. In addition, we observed using B-mode ultrasound a very different behavior between tasks: an increase in muscle length and an horizontalization of its fibers during Valsalva maneuver, while a shortening of the muscle and a downward tilt of its fibers occurred during contractions. This supports the fact that we effectively measure the muscle properties in two different conditions. These results highlight the large lengthening of the LAM during a Valsalva maneuver that significantly increase its stiffness in a similar manner than during contractions. The increase in stiffness is probably much larger during childbirth explaining the risk of muscle trauma. Lastly, these interpretations about the value of the shear modulus of the LAM at contraction should be carefully considered regarding the poor reliability of such a measure, the difficulty to standardize the task and to be sure that the contraction is maximal.

The comparison with the literature on LAM elastic behavior remains complex because various methods do not provide values in the same metrics. We cannot compare our results about the LAM viscoelastic properties to biomechanical studies on cadaveric tissues because in these works, researchers aim to identify the level of strain for which damage occurs and not the intrinsic elastic properties. Our results are not comparable to study involving the use of vaginal speculum as an elastometer or vaginal probe because it measures a torque or a force applied on the device and recorded by force sensor, which is not a direct quantitative assessment of elastic properties as using elastography^9,10^. We can compare our data to other elastography studies. A more direct comparison can be done with the study of Tang et al. using SWE, reporting a 28 kPa shear modulus for the LAM at rest (versus 22 in our study) and 57 kPa during Valsalva maneuver (vs 45 in our study). Therefore, Tang et al. report a little stiffer LAM but in a very different population with a mean age of 56 years versus 23 in our study^30^. Finally, as done in our previous study^12^, we can compare our results to a study of Silva et al. that calculated the elastic properties of the pubovisceral muscle using inverse finite element with three models. They reported a shear modulus of 78 ± 44 kPa with the first one, 80 + /- 48 kPa with the second one and 62 + /- 46 kPa with the last one^32^. Silva et al.’s reported a stiffer LAM muscle than in the present study, but values remained in the same range and very different methods were used. Taken these comparisons all together, the range of values reported in the present study seems consistent with the literature.

The LAM appears to be much stiffer than the peripheral muscles*.* Indeed, we reported an SM of 22 kPa for the LAM at rest, whereas it has been reported to be between 2 and 5 kPa for peripheral muscles of both the upper and lower limbs^20^. This difference may be primarily associated with differences in the intrinsic structure of these muscles, since the LAM mainly consists of type 1 muscular fibers (mainly involved in prolonged effort), whereas peripheral limb muscles mainly consist of type 2 muscle fibers^33^. Another hypothesis could be that measurements in the LAM were performed near the muscle’s pubic insertion, whereas measurements for peripheral muscles were mainly performed in the middle of the muscle with distance to its insertions^20^. Furthermore, even if measurements were performed without the Valsalva maneuver or subjective maximal contraction and in a lithotomy position, there is always a constraint applied by abdominal pressure into pelvic floor muscles that could never be fully removed in in vivo conditions.

Our results showed that SWE is a reliable tool to investigate the elastic properties of pelvic floor muscles in vivo. This offers interesting prospects for research that will aim to improve our knowledge of the pathological processes associated with obstetric perineal trauma and/or PFD occurrence. A prospective study is ongoing in a pregnant women cohort that investigates changes in the elastic properties of pelvic floor muscles and peripheral muscles during pregnancy by using the same protocol as that described in this paper^11^.

## Conclusion

Ultrasound SWE is a reliable tool to investigate LAM elastic properties at rest and during the Valsalva maneuver, but the present study failed to perform reliable measurements during perineal subjective maximal contraction. This technology might be useful to improve our knowledge of the pathological processes associated with obstetric perineal trauma and/or pelvic floor disorders.

## Supplementary Information

Supplementary file.

Supplementary Video 1.

## Abbreviations

BMI: Body Mass Index
CV: Coefficient of variation
ICC: Intraclass correlation coefficient
LAM: Levator ani muscle
PFD: Pelvic floor disorders
SEM: Standard error of measurement
SM: Shear modulus
SWE: Shear wave elastography
