# Supplementary material for: Transperineal ultrasound shear-wave elastography is a reliable tool for assessment of the elastic properties of the levator ani muscle in women
Source: Sci Rep. 2021 Jul 30;11:15532. doi: 10.1038/s41598-021-95012-8 (PMC8324884; doi:10.1038/s41598-021-95012-8)
Supplement: Supplementary file 1 — Supplementary file. [file 41598_2021_95012_MOESM1_ESM.docx]

**Title**: Transperineal ultrasound shear-wave elastography is a reliable tool for assessment of the elastic properties of the levator ani muscle in women

**Authors:**

Bertrand Gachon

Xavier Fritel

Fabrice Pierre

Antoine Nordez

**Supplementary material 1**: Video clip of an ultrasound shear wave elastography assessment of the right levator ani muscle during the Valsalva maneuver
